# Multiple sclerosis and stroke: a systematic review and meta-analysis

**DOI:** 10.1186/s12883-019-1366-7

**Published:** 2019-06-24

**Authors:** Ye Hong, Huai Rong Tang, Mengmeng Ma, Ning Chen, Xin Xie, Li He

**Affiliations:** 1 Department of Neurology, West China Hospital of Sichuan University, Wainan Guoxue Xiang #37, 610041 Chengdu, People’s Republic of China; 2 Department of Health Management Center, West China Hospital of Sichuan University, Chengdu, China; 3 Department of Neurology, The General Hospital of Western Theater Command, Chengdu, China

**Keywords:** Multiple sclerosis, Stroke, Ischemic stroke, Prevention

## Abstract

**Background:**

Multiple sclerosis (MS) and stroke are two common causes of death and disability worldwide. The relationship between these two diseases remains unclear. Effective early preventative measures and treatments are available to reduce the morbidity and mortality of acute stroke. The objectives of our systematic review are to estimate the risk of stroke in patients with MS and to collate related studies to draw preliminary conclusions that may improve clinical practice.

**Method:**

Relevant studies were systematically searched in MEDLINE, Embase, the Chinese Biomedical Literature Database (CBM), the China National Knowledge Infrastructure and the VIP database of Chinese periodicals from January 1983 to May 2017, with no restrictions on language. Patients included in this review were adults who suffered from MS. Review Manager 5.3 software program was used to pool data and calculate the risk ratio (RR) and its 95% confidence interval (CI). We also performed heterogeneity and sensitivity analyses and evaluated bias in the meta-analysis.

**Results:**

Nine studies including more than 380,000 participants that met our inclusion criteria were incorporated into the meta-analysis. During different follow-up periods, patients with MS had an increased risk of any type of stroke [RR = 3.48, 95% CI (1.59, 7.64), *P* = 0.002 for 1 year; RR = 2.45, 95% CI (1.90, 3.16), *P* < 0.00001 for 10–13 years]. The total prevalence of stroke (any type) in patients with MS exceeded expectations compared to different groups [Comparing with general veteran: RR = 2, 95% CI (1.19, 3.38), *P* = 0.009. Comparing with general population: RR = 2.93, 95% CI (1.13, 7.62), *P* = 0.03]. Furthermore, ischemic stroke was particularly more common in the MS population than in people without MS [RR = 6.09, 95% CI (3.44, 10.77), *P* < 0.00001].

**Conclusion:**

Compared with the general population, people with MS have an increased risk of developing any type of stroke and ischemic stroke in particular. Consistent results were obtained from patients of different sexes and age groups. Preventative measures and treatments should be administered at earlier time points to improve patient outcomes.

**Electronic supplementary material:**

The online version of this article (10.1186/s12883-019-1366-7) contains supplementary material, which is available to authorized users.

## Background

Multiple sclerosis (MS), an autoimmune disease of the central nervous system (CNS), is one of the most common causes of neurological disabilities in young adults and is recognized to be a pathological consequence of immune-mediated inflammation, demyelination and subsequent axonal damage [1]. Most patients experience a gradual loss of visual, motor and sensory functions. Standardized definitions for the clinical courses of MS include relapsing-remitting, secondary progressive, primary progressive, and progressive relapsing [2]. According to the World Health Organization (WHO), the prevalence of MS varies in different countries, reaching approximately 0.08% in Europe, and the highest prevalence is observed in women [3]. In Southeast Asia, the sex ratio (female vs. male) ranges from 0.7 (India) to 9.0 (China). The average age of people with MS ranges from 10 to 60 years. Meanwhile, women experience a peak risk at ages ranging from 40 to 60 years [4–6]. Stroke, the leading cause of death and disability, is the third most common disease worldwide [7]. Patients are mainly middle-aged and elderly people. The quality of life of patients who have experienced a stroke is substantially affected. People with multiple sclerosis may suffer from more severe symptoms if they also experienced a stroke and ischemic stroke. The increasing risk of cerebral ischemia in people with MS will further aggravate the disease burden. A Sweden study reported mortality rates and event risk ratios (RRs) of ischemic stroke in people with MS [8]. People with MS were more likely to develop ischemic stroke. However, the association between MS and stroke remains controversial. One study observed a greater incidence of ischemic stroke in non-MS patients[9]. Therefore, the occurrence of stroke and the potential burden of disease must be reduced by definitively determining the correlation between MS and stroke to implement more timely and effective preventative and therapeutic measures.

A systematic review was conducted to determine the incidence and prevalence of cardiac and cerebrovascular diseases in people with multiple sclerosis [10]. However, some newly published original studies might report new findings. Additionally, as mentioned above, differences between sexes and age groups might exert substantial effects on the pathology of MS and stroke. However, the review did not show any analysis of groups stratified by sex or age. Thus, for better implementation of strategic interventions and determination of the prognosis, a systemic analysis of the correlation between MS and stroke is needed. Hence, we conducted this meta-analysis to evaluate and compare the different risks of stroke and ischemic stroke in people with MS and non-MS populations in more detail, including differences in groups stratified by sex and age, thus providing more reliable evidence for clinical practice.

## Methods

We strictly followed the MOOSE (Meta-Analysis of Observational Studies in Epidemiology) guidelines throughout the design, implementation, analysis and reporting processes in this study [11].

### Search strategy and information sources

Studies included in MEDLINE, Embase, Chinese Biomedical Literature Database (CBM), the China National Knowledge Infrastructure (CNKI) and the VIP database of Chinese periodicals that were published from January 1983 to May 2017 were searched to identify potentially relevant observational studies. Cohort, case-control and cross-sectional studies were all included. Search terms were established with the subject headings and keywords “Stroke or Ischemic Stroke or Hemorrhage Stroke or Intracranial Embolism or Thrombosis or Cerebrovascular incident or Cerebrovascular Disorders” and “Multiple sclerosis”.

### Study selection

**Fig. 1:**
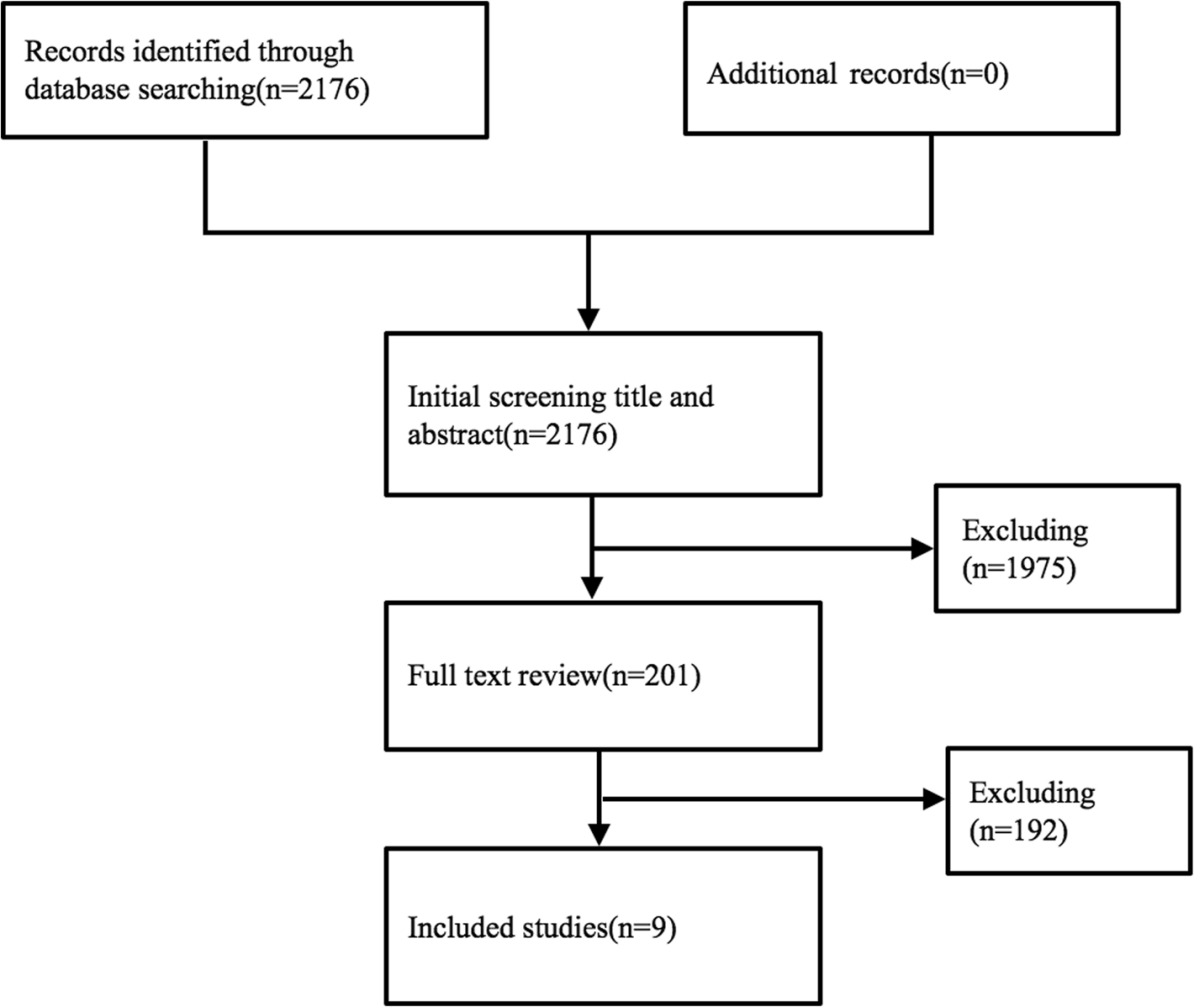
Process used to select the included studies

Two qualified investigators independently assessed the eligibility of the identified publications; discrepancies were resolved by discussion. Broad inclusion criteria were used for the studies, with no limitations on language, stroke type or stroke phase. We first performed an initial screen of titles or abstracts to assess potential relevance. Afterwards, we obtained relevant full-text articles, reevaluated their eligibility and determined their final inclusion or exclusion. Studies were included if they were published between January 1983 and May 2017; reported data from an original, peer-reviewed observational study or from an observational source; were conducted in a group of people with MS and a matched control population and used definite diagnostic criteria for MS and stroke. We excluded studies that used an ambiguous definition of MS or stroke, were unpublished findings or published as reviews, editorials, duplicate citations, nonhuman studies or abstracts with unavailable data. Corresponding authors of the included studies were contacted electronically at least twice to obtain any missing data. When studies had been published more than one time on the same topic and theme, only the most recent study was selected to extract data, others were listed and analyzed as similar studies. Additionally, we reviewed the references and full text of all selected studies. Based on the pre-specified selection criteria, we excluded 1776 studies after the initial screen. One hundred eighty-one additional studies were excluded after a review of the full text review for several major reasons: studies were not conducted in groups of MS and matched control populations and reported relative medical outcomes of stroke; studies of transient stroke-like syndromes only or silent infarcts, in which the temporal relationship between MS and stroke is difficult to determine; and the timing of the diagnosis of MS with respect to the development of stroke was difficult to determine in the study. The selection process is shown in Fig. 1.

### Data extraction and quality assessment

A standardized data collection form including the authors’ names, publication year, study country (regions), study design, number of participants, participants’ ages, sexes and sex ratios, follow-up periods, study periods and quality scores was used to extract essential information (Additional file 1). The quality of each study was critically evaluated by two investigators using the Newcastle-Ottawa Quality Assessment Scale and included the following variables: appropriateness and reporting of inclusion and exclusion criteria, exposure assessment, outcome assessment, control of confounding variables, and evidence of bias. Two investigators provided yes or no responses to nine questions for each article. Values of 0 or 1 were assigned to these variables, with 1 representing the best score. The total score was 9. Quality scores greater than 7 were considered high quality.

### Statistical analysis

Review Manager software version 5.3 was used with a fixed-effect model to pool data, and a forest plot was generated to calculate risk ratios (RRs) and their 95% confidence intervals (CIs). The chi-square test was performed to test the hypothesis (Z distribution, *P* < 0.05 was considered statistically significant). I^2^ statistics were used to assess the heterogeneity among studies (values less than 50% indicate acceptable heterogeneity and values greater than 50% indicate substantial heterogeneity). When heterogeneity was unable to be readily explained, one analytical approach is to incorporate heterogeneity into a random-effects model. For any particular set of studies in which heterogeneity is present, a confidence interval including the random-effects pooled estimate is wider than a confidence interval including a fixed-effects pooled estimate. Therefore, a random-effects model and subgroup analysis were performed if the source of heterogeneity was unable to be determined. A sensitivity analysis was performed to predict stability and bias (including publication bias, selective reporting within studies, etc.). A meta-analysis was conducted if sources were available from a minimum of two studies.

## Results

### Literature search and quality assessment

Nine observational studies fulfilled our inclusion criteria and were included in the meta-analysis (the selection process is shown in Fig. 1) [8, 12–19]. Four of these studies were conducted in Europe (44.5%) [14, 17–19], three in North America (33.3%) [8, 15, 16], and 2 in Asia (22.2%) [12, 13]. Two were limited to Asian Chinese subjects [12, 13], and the other studies examined North American and European subjects (Caucasian population) [8, 14–19]. Studies were conducted from 1972 to 2012. One of these studies was a case-control and cohort study [14], while three were cross-sectional studies [13, 15, 16] and five were cohort studies [8, 12, 17–19]. The outcome measures of one study were determined based on a “yes” response to questions asking whether the participants had ever been told by a health professional that they had experienced a stroke [16], while the outcomes in other studies were determined based on international classification codes [8, 12–15, 17–19]. The data were all obtained from national (regional) medical-related databases with a large number of participants. The number of people with MS ranged from 898 to 15,684, and 69,805 participants were diagnosed with multiple sclerosis. The minimum number of non-MS subjects was 4490 [13], and the total number was 316,361. We attempted to contact the corresponding author of one study to obtain missing data regarding the maximum number of control subjects (over 9 million) but received no reply [17]. Follow-up periods ranged from one year to approximately 30 years. Participants’ ages ranged from 15 to 84 years, and people of almost all ages were examined. The sex ratio (male vs. female) was 1:3 in 2 studies [8, 12] and 1:2 in 5 studies [14, 15, 17–19]. One study was restricted to males [16], and one did not specify its sex ratio [13]. Proportions of stroke incidence were the main outcome measures for five studies [8, 12, 17–19]. An Asian study also reported the 5-year incidence [12]. Three studies regarded stroke prevalence as the main outcome measure [13, 15, 16]. One study calculated the number of patients who received a new diagnosis of stroke after MS onset and treated the result as the main outcome measure [14]. Four studies reported the risk of ischemic stroke in people with MS [8, 12, 15, 18]. A quality assessment was conducted, and scores ranged from 5 to 9. Five studies were categorized as high quality [8, 12, 17–19]. The main reasons for the lower quality scores of the other 4 studies [13, 14, 16, 17] were weak representation and substantial loss to follow-up.

### The incidence of any type of stroke

**Fig. 2:**
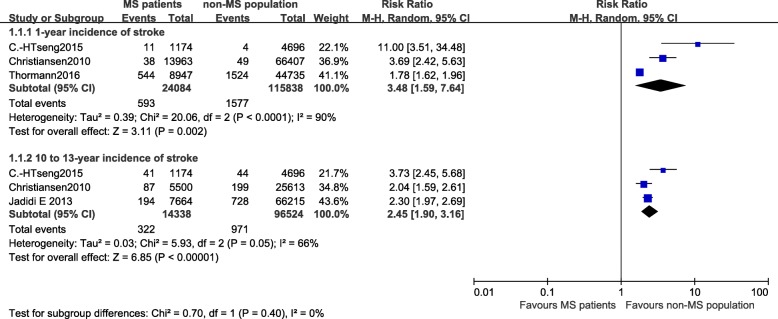
Forest plot of the incidence of stroke in patients with MS and the non-MS population after 1 year and 10–13 years

The incidence of any type of stroke, including ischemic stroke, hemorrhagic stroke and transient ischemic attack (TIA), ranged from 2.53% [19] to 2.85% [18], and the incidence of ischemic stroke ranged from 1.22% [18] to 3.49% [12]. The results for any type of stroke are shown first, and studies were divided into two groups to conduct a meta-analysis because of the differences in follow-up periods in several studies (Fig. 2). The fixed-effect meta-analysis revealed substantial heterogeneity, and the heterogeneity was unable to be readily explained; consequently, a random-effects model was used instead.

#### One-year follow-up

Three studies reported the 1-year incidence of any type of stroke [12, 14, 18]. The summary incidence estimate for these studies was 3.48 [95% CI (1.59, 7.64), *P* = 0.002]. The heterogeneity among these studies was high (I^2^ = 90%).

#### Ten- to thirteen-year follow-up

The three longest follow-up periods were 10, 12 and 13 years [12, 18, 19]. People with MS had a higher risk of stroke than non-MS patients during these 10- to 13-year follow-up periods [RR = 2.45, 95% CI (1.90, 3.16), *P* < 0.00001]. Heterogeneity was moderate (I^2^ = 66%) and may have been triggered by differences in the study population, study design and stroke diagnostic criteria.

Furthermore, the results of another study were consistent with our conclusions [17]. During the first year after MS onset, people with MS were more likely to develop an ischemic stroke [RR = 2.02, 95% CI (1.90, 2.14)] and hemorrhagic stroke [RR = 2.65, 95% CI (2.27, 3.08)] than non-MS populations. After 1–5 years, the risks of ischemic stroke [RR = 1.50, 95% CI (1.46, 1.55)] and hemorrhagic stroke decreased [RR = 1.83, 95% CI (1.69, 1.98)]. At 10 years after MS onset, the risk decreased further [ischemic stroke: RR = 1.29, 95% CI (1.23, 1.35); hemorrhagic stroke RR = 1.47, 95% CI (1.31, 1.65)] [17]. Thus, people with MS were more likely to be hospitalized for a stroke than non-MS population. Additionally, during the course of the study by Christiansen CF, the incidence of stroke was particularly high among people with MS [incident rate ratio (IRR) = 1.23, 95% CI (1.10, 1.38)] [18]. However, since the definition of MS relapses occasionally mimics TIA, an exact evaluation of the TIA risk in a population with MS relapses was difficult. We should be aware of the accuracy of the diagnosis, and the heterogeneity might be affected by misdiagnosis.

### The prevalence of any type of stroke

**Fig. 3:**
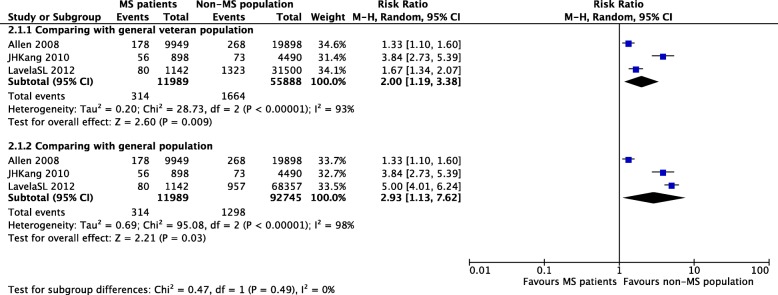
Forest plot of the prevalence of stroke in patients with MS and the non-MS population in studies using different comparison populations

Three studies reported the prevalence of any type of stroke [13, 15, 16]. The prevalence ranged from 0.4% [13] to 7.0% [16]. One study compared three groups, namely, male veterans with MS, male veterans without MS and the general population; we analyzed the data accordingly [16]. The prevalence of any type of stroke appeared to be higher in male veterans with MS than in male veterans without MS [RR = 2.00, 95% CI (1.19, 3.38), *P* = 0.009] and the general population [RR = 2.93, 95% CI (1.13, 7.62), *P* = 0.03]. Heterogeneity was substantial in both comparisons (I^2^ = 93 and 98%), possibly due to the use of different study populations (Fig. 3). We should be aware of the accuracy of the diagnosis, and the heterogeneity might be affected by the misdiagnosis of MS and TIAs as well.

### The occurrence of ischemic stroke

**Fig. 4:**
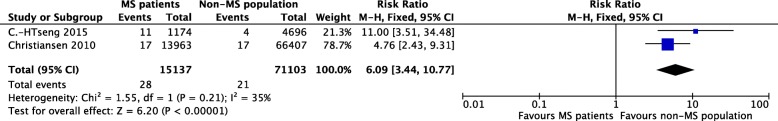
Forest plot of the incidence of ischemic stroke in patients with MS and the non-MS population

Regarding ischemic stroke, two studies mentioned the incidence of ischemic stroke after a 1-year follow-up [12, 18]. The summary incidence estimate for these two studies was 6.09 [95% CI (3.44, 10.77), *P* < 0.00001]. Heterogeneity was not substantial (I^2^ = 35%), perhaps as a result of differences in race, sex ratio and number of included participants (Fig. 4).Moreover, an Asian study revealed an incidence of ischemic stroke of 9.96/1000 person-years in people with MS and 0.90/1000 person-years in a non-MS population [12]. Moreover, the 5-year incidence of ischemic stroke was 8.12/1000 person-years in people with MS and 1.48/1000 person-years in non-MS population. The summary incidence estimate was defined as an adjusted hazard ratio (HR) = 12.1 for 1 year and adjusted HR = 4.69 for 2–5 years compared with the non-MS population. In addition, people with MS aged ≤40 years had a higher risk of ischemic stroke [HR 12.7, 95% CI (3.44, 46.7)] [12]. The influence of MS on the risk of stroke was particularly important in younger patients (data not shown). Vascular disorders of the CNS were reported to be more common in the MS cohort than in the comparison cohort [RR = 3.6, 95% CI (3.5, 3.8)], with the highest event rate ratio (ERR) for ischemic stroke (RR = 3.8, 95% CI (3.5, 4.2) [8]. According to another study, people with MS are more likely to be hospitalized for ischemic stroke [OR = 1.66, 95% CI (1.33, 2.09)] than a matched non-MS cohort, as determined by comparing mortality rates and event rates with matched non-MS cohorts from the United States Department of Defense (DoD) database [15]. Findings from these studies were fairly consistent.

## Subgroup analyses

### The incidence of any type of stroke

#### Age

**Fig. 5:**
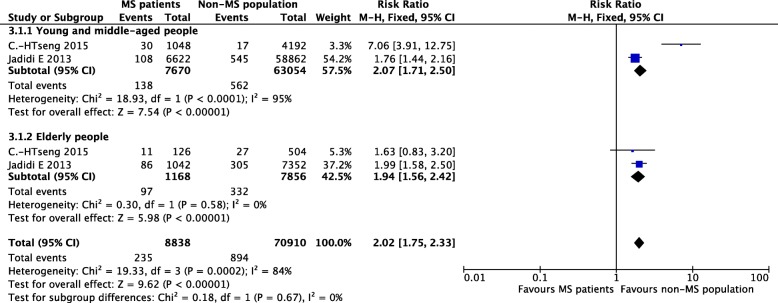
Pooled estimate of subgroups stratified by age

Of the three studies that compared the incidence of stroke in different age groups [12, 18, 19], two reported approximate follow-up periods [12, 19]. Participants were divided into two age groups: a younger group aged < 55 or 60 years and an elderly group aged > 55 or 60 years. The summary incidence estimate was 2.02 [95% CI (1.75, 2.33), *P* < 0.0001]. The overall heterogeneity was substantial (I^2^ = 84%) and higher than the analysis without subgroups [19]. The heterogeneity between subgroups was zero (I^2^ = 0), indicating that the age differences were not related to the substantial heterogeneity (Fig. 5).

#### Sex

**Fig. 6:**
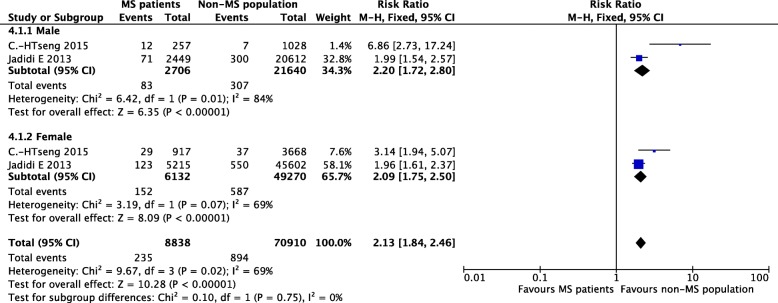
Pooled estimate of subgroups stratified by sex

People with MS were mostly women aged less than 40 years [20]. Of the two studies that reported different risks of stroke between the two sexes, the sex ratio was 1:3 (male vs female) in an Asian study [12] and 1:2 in a Swedish study [19]. The summary incidence estimate was 2.13 [95% CI (1.84, 2.46), *P* < 0.00001]. The heterogeneity was moderate (I^2^ = 69%) and very similar to the heterogeneity of the analysis without any subgroups. Additionally, the heterogeneity of each subgroup was zero (I^2^ = 0), indicating that different sex ratios were not associated with the moderate heterogeneity described above (Fig. 6).

### The prevalence of any type of stroke in comparisons of different subpopulations

**Fig. 7:**
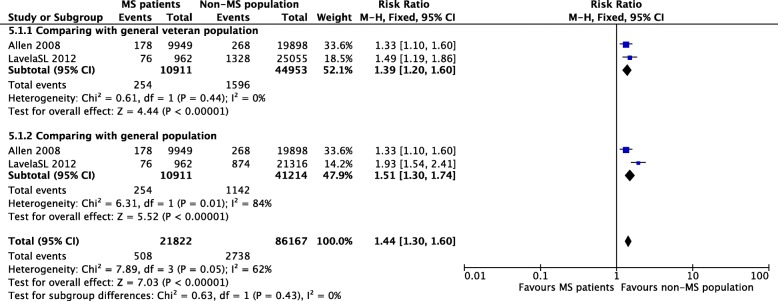
Pooled estimate of subgroups stratified by different comparison populations

Of the two cross-sectional studies that reported the prevalence of stroke in middle-aged and elderly people [15, 16], one study included two different cohorts as two comparison groups [16]. The summary incidence estimate was 1.44 [95% CI (1.30, 1.60), *P* < 0.00001]. The heterogeneity was moderate (I^2^ = 62%) and was significantly smaller than the analyses without subgroups (I^2^ = 93 and 98%). The heterogeneity of each group was unremarkable (I^2^ = 0), indicating that the substantial heterogeneity in prevalence was associated with the use of different cohorts in comparison groups (Fig. 7).

### Confounding factors

#### The onset of MS

Thormann et al. reported a decreased risk of stroke in people with MS prior to the clinical onset of MS, but an increased incidence of cerebrovascular comorbidity was observed in people with MS after the clinical onset [14]. In the aforementioned study, the duration of MS, but not the age of the patients, might trigger the increased risk of stroke itself [14]. The onset of MS should be regarded as an important confounding factor and is worthy of extensive discussion.

#### Risk behaviors

Smoking, hypertension, diabetes, hypercholesterolemia and obesity are well-known independent risk factors for cerebrovascular diseases. Allen et al. reported a significantly lower prevalence of hypertension, diabetes, hypercholesterolemia and obesity in people with MS [15]. However, other studies reported completely inconsistent results for these comorbidities in both Western countries and East Asia [12, 13, 16, 19]. Additionally, smoking, a cerebrovascular risk behavior that is known to be common in people with MS, should be discussed as a potential confounder. Unfortunately, the information on smoking at the individual level was not available in any of the included studies. In addition, other confounding variables, including alcohol consumption, diet, overweight and physical activity, were unable to be determined. The analysis in our study might be affected by the bias in the included studies. We should interpret the results with caution.

#### Severe comorbidities and infections

Three of the included studies described the occurrence of several systematic comorbidities in people with MS compared to controls [8, 13, 19]. The variables analyzed were not completely consistent among studies. Jadidi et al. did not observe a significant difference in the risks of chronic obstructive pulmonary disease, renal failure and liver diseases [19]. A study of ethnic Chinese people revealed that people with MS were more likely to be diagnosed with systemic lupus erythematosus, epilepsy, depression, peripheral vascular disorders, deficiency anemias, rheumatoid arthritis, fluid and electrolyte disorders, migraines, dementia, psychoses, tuberculosis, inflammatory spondylopathies, hypothyroidism, peptic ulcer and pulmonary circulation disorders [13]. This study did not observe a significant difference in the risk of developing renal failure, consistent with the results reported by Jadidi [19]. Another observational study focused on the rate of infections among the MS cohort compared with the non-MS cohort [8]. Among infections, the highest ERRs were observed for sepsis, opportunistic infections and urinary tract infections. Moreover, the ERRs decreased with increasing age, except for urinary tract infections, which increased with age. The presence of severe comorbidities might interfere with ordinary functioning, leading to reduced mobility, particularly in elderly patients. For example, immune-mediated diseases (IMDs) have been reported to promote the formation of atherosclerosis. Some IMDs are associated with an increased risk of stroke. Thus, confounding factors should also be analyzed when possible to reach exact conclusions.

## Sensitivity analysis

Of the studies that reported the incidence of any type of stroke, quality scores ranged from 6 to 9. A sensitivity analysis was performed after excluding the lower quality study [14]. The sample size was changed, and the combined effect was 4.32 [95% CI (2.92, 6.38), *P* < 0.00001]. The heterogeneity was moderate (I^2^ = 68%) and smaller than before the exclusion of the low quality study, which had some bias [14]. Of the studies that reported the prevalence of any type of stroke, quality scores ranged from 5 to 6. Two studies were of low quality [13, 16]. The summary prevalence estimate was not typically changed when the study by Lavela was excluded, but changed markedly when the study by Kang was excluded. The heterogeneity was moderate (I^2^ = 59%) when the study by Kang was excluded, indicating that study clearly exhibited bias [13, 16].

## Discussion

MS, the most common cause of neurological disability among young adults, is generally believed to be a chronic inflammatory demyelinating disease, which has a median prevalence of 80/100,000 people in Europe. Inflammation in autoimmune diseases can damage the normal physiological function of the endothelium, accelerate the process of atherosclerosis, and increase the risks of cerebrovascular diseases, particularly ischemic stroke [21–23]. Furthermore, the fatty myelin sheaths around the axons are damaged in the CNS, leading to demyelination, remyelination, axonal loss, gliosis and neurodegeneration [3]. The condition may persist for months to years and increase the risk of arterial atherosclerosis [24, 25]. Of the studies comparing the incidence or prevalence of stroke and ischemic stroke in the MS population to a comparison population [8, 12–19, 26–28], most reported increased risks of stroke and ischemic stroke in the incident MS population compared with an age- and sex-matched cohort from the general population. The incidence of stroke decreased with the onset of MS but was still higher than in the non-MS population. However, studies from Fleming et al. were restricted to hospitalized patients with prevalent MS aged greater 65 years and found that people with MS were less likely to have cerebrovascular diseases[10, 29]. We proposed explanations for the inconsistencies, including an artifact of their dataset, which only included up to 5 diagnoses per discharge, and the restriction of the analysis to elderly people, because people with MS and comorbidities might be more likely to die before reaching 65 years old, as well as a protective effect of MS or its treatment. The contradictory results remind us of our gaps in the knowledge of MS and methodological limitations of our study.

Heterogeneity is potentially attributed to several factors, such as (i) the use of different cohort selection criteria. In this systematic review, we identified several population-based studies that evaluated the comorbidities and vascular risk factors in people with MS. However, the incidence, prevalence and associated rates of vascular diseases vary substantially worldwide[30, 31]. In an Asian study that mainly consisted of ethnic Chinese subjects [13], the results for some comorbidities were not completely consistent with previous studies conducted in Western countries[32, 33]. Additionally, ethnic and geographic differences also affect the MS prevalence, clinical presentation, and courses [34]. Two Danish studies observed a nearly 30% increased risk of mortality from vascular disabilities in people with MS, while another South Wales study only observed a 6% increased risk compared to matched controls [35]. Of the studies comparing the incidence or prevalence of ischemic stroke and any type of stroke in people with MS to comparison groups, two studies from North America contained different proportions of white non-Latino, black non-Latino, Latino, and other ethnic groups [15, 16]. In addition, sexes and occupations might also account for the heterogeneity because these two factors were restricted in the selection criteria of most studies. (ii) The definitions and diagnoses of MS and stroke also differed. During the last 20 years, the diagnostic criteria for MS have changed[36–40]. Our included studies were conducted from 1972 to 2012, and thus differences in diagnostic criteria existed. Several studies were not completely comparable due to the change in diagnostic criteria. For most of our included studies, International Classification of Diseases (ICD) codes were the main criteria for diagnosing stroke and ischemic stroke. TIA, occlusion and stenosis of the peripheral cerebral artery, and intracerebral hemorrhage were also reported. (iii) Follow-up periods and age distributions varied. Follow-up periods ranged from one year to approximately 30 years. Although we grouped studies according to their follow-up periods and analyzed them accordingly, the discordant follow-up periods might contribute to the heterogeneity in the summary incidence estimate. Age is an important factor to consider in cerebrovascular diseases, but a standardization of the estimates to account for differences in the age of the participants was lacking in some studies[41–43].

Based on this systematic review, our conclusion that stroke and ischemic stroke occur more frequently in patients with MS should be interpreted with caution due to the gaps in our knowledge of MS and methodological limitations. People with MS displayed an elevated risk of stroke over different periods. A definite conclusion about the most common subtypes of stroke occurring in people with MS was not reached in a recent study. A study from Finland showed that 6 patients in the MS cohort had experienced a stroke, 5 of whom had experienced an acute ischemic stroke in the large vessels and one of whom had experienced a TIA with a few hours of aphasia [27]. Further studies are needed. Some potential common risk factors for MS and stroke are listed below.

First, obesity in childhood and early adolescence may accelerate the development of MS and increase the intima-media thickness, which is correlated with coronary artery diseases and represents a predictor of stroke [26, 44].

Second, ischemic stroke may also be induced by T-cells specific to Epstein-Barr virus during the inflammatory reaction in atherosclerotic plaques, as well as low levels of vitamin D, a possible risk factor for MS [45–49]. Brain-related symptoms observed in people with MS may be caused by vascular epithelial cells, and the demyelination of neurons may lead to a series of ischemic changes in the early phase [50, 51].

Additionally, people with MS and patients who have experienced a stroke have some unhealthy habits. Individuals with MS are more likely to smoke than individuals without MS. Most individuals with MS also have low levels of physical activity [52].

Furthermore, MS treatments may also increase the risk of vascular diseases [53]. Systemic glucocorticoids may increase the risk of cerebrovascular and cardiovascular diseases[54]. High-dose glucocorticoid use has been reported to increase the risks of myocardial infarction and stroke. A positive correlation between cardiovascular risk factors and the use of disease-modifying therapies, such as interferon and glatiramer acetate, has been observed [55, 56].

Specifically, pregnancy, a special cause of ischemic stroke in women, may account for some stroke incidents in young female patients[57, 58]. Thus, these relative risk factors, including vitamin D deficiency, Epstein-Barr virus, childhood obesity, unhealthy habits and pregnancy might partially contribute to the epigenetic mechanisms of these two diseases. Based on our findings, clinicians should focus on cerebral ischemic changes in people with MS, particularly in female patients during the child-bearing period, and cautiously prescribe MS clinical treatments to prevent the incidence of stroke.

Our review also has some limitations. For example, the included studies lacked independent analyses of lifestyle factors, social status, education and other factors that might affect the progression of the disease. A more specific discussion of the risk of hemorrhagic stroke is needed. Besides, individuals with MS presenting an exacerbation may exhibit stroke-like symptoms, which can be misclassified as a cerebrovascular event [14, 18, 59]. Moreover, patients with a clinical isolated demyelination episode can be diagnosed as MS now following the newly criteria announced in the year 2017 [60]. However, since most of our included studies were conducted before 2017, patients with a single event were potentially went undiagnosed. Considering of this, we should be more cautious about the conclusions. Thus, the quality of these included studies might be affected. Despite these limitations, our findings are still meaningful for clinical practice. Future research should focus on other social and environmental factors and on the early treatment and prevention of stroke in people with MS.

## Conclusions

Our systematic review is intended to estimate the incidence and prevalence of ischemic stroke and of any type of stroke among people with MS. This study analyzes age and gender, two possible risk factors, to a greater extent than in previous systematic reviews on the subject [7]. However, since many gaps in our knowledge of the association between MS and stroke remain, the statistical analysis and relative RRs reported here should be regarded very prudently. We cautiously conclude that people with MS are generally more likely to develop stroke, particularly ischemic stroke, than non-MS individuals.

## Additional file

Additional file 1: Characteristics of the included studies. (DOCX 19 kb)

## Data Availability

All data generated or analyzed during this study are included in this published article and its supplementary information files.

## Abbreviations

CBM: Chinese Biomedical Literature Database
CI: confidence interval
CNKI: China National Knowledge Infrastructure
CNS: Central nervous system
ERR: Event rate ratio
HR: Hazard ratio
ICD: International Classification of Diseases
IMDs: Immune-mediated diseases
IRR: Incident rate ratio
MOOSE: Meta-Analysis of Observational Studies in Epidemiology
MRR: Mortality rate ratio
MS: Multiple sclerosis
NA: Not applicable
RR: Risk ratio
TIA: Transient ischemic attack
WHO: World Health Organization
