# Supplementary material for: Multiple sclerosis and stroke: a systematic review and meta-analysis
Source: BMC Neurol. 2019 Jun 24;19:139. doi: 10.1186/s12883-019-1366-7 (PMC6591845; doi:10.1186/s12883-019-1366-7)
Supplement: Supplementary file 1 — Characteristics of the included studies. (DOCX 19 kb) [file 12883_2019_1366_MOESM1_ESM.docx]

| Study ID | Region | Study Type | Sex  (Male/  Female) | Mean Age／Age Range | Follow up  Time  (years) | N*of  study population  (MS/Non-MS ) | N*of  study population  with stroke  (MS/Non-MS) | Quality Score | Study  Period |
| --- | --- | --- | --- | --- | --- | --- | --- | --- | --- |
| Allen  2008 | The United States | Cross-sectional Study | 1:2 | 40-84 | NA | 9949/  19,898 | 178/  268 | 6/9 | 1988~  2002 |
| Thormann  2016 | Denmark | Cohort Study/  Case-control Study | 1:2 | 34.68 (MS patients) | 1～35 | 8947/  44,735 | 544/  1524 | 6/9 | 1977~  2012 |
| Bengt  Zoller  2012 | Sweden | Cohort Study | 1:2 | No  Age  limitations | 10+ | 10,384/  Over 9million  (no exact details) | 237/  494,303 | 8/9 | 1987～2008 |
| C.H  Tseng 2015 | Taiwan, China | Cohort Study | 1:3 | 37.1±  13.8  (MS patients)  37.1±  13.9  (Non  MS  population) | 1~13 | 1174/  4696 | 42/44 | 8/9 | 1997~  2010 |
| Capkun  2015 | The United States | Cohort Study | 1:2 | 46±  11.7 | 1.9～5.9 | 15,684/  78,420 | 742/  920 | 7/9 | 2006~  2011 |
| Christiansen  CF  2010 | Denmark | Cohort Study | 1:2 | No  Age  limitations | 2~30 | 13,963/  66,407 | 398/  2318 | 9/9 | 1977~  2006 |
| Jadidi E  2013 | Sweden | Cohort Study | 1:2 | NA | 11 | 7664/  66,215 | 194/  728 | 7/9 | 1987~  2009 |
| JH.  Kang 2010 | Taiwan, China | Cross-sectional Study | NA | >15 | 1 | 898/  4490 | 56/73 | 5/9 | 2006~  2007 |
| Lavela SL  2012 | The United States | Cross-sectional Study | Males only | 60.8  (MS patients)  59.1  (Non-MS population) | 1 | 1142/  31,500 | 80/1323 | 5/9 | 2003~  2004 |

**Additional file 1: characteristics of included studies.**

**N*:number**

**NA: Not applicable**
